# BipC, a Predicted *Burkholderia pseudomallei* Type 3 Secretion System Translocator Protein with Actin Binding Activity

**DOI:** 10.3389/fcimb.2017.00333

**Published:** 2017-07-19

**Authors:** Charles W. Vander Broek, Nurhamimah Zainal Abidin, Joanne M. Stevens

**Affiliations:** The Roslin Institute and Royal (Dick) School of Veterinary Studies, University of Edinburgh Scotland, United Kingdom

**Keywords:** BipC, SipC, T3SS, effector, translocator, actin binding, *Burkholderia pseudomallei*, melioidosis

## Abstract

*Burkholderia pseudomallei* is an intracellular bacterial pathogen and the causative agent of melioidosis, a severe disease of humans and animals. Like other clinically important Gram-negative bacteria, fundamental to *B. pseudomallei* pathogenesis is the Bsa Type III Secretion System. The Bsa system injects bacterial effector proteins into the cytoplasm of target host cells subverting cellular pathways for the benefit of the bacteria. It is required for invasion of non-phagocytic host cells, escape from the endocytic compartment into the host cell cytoplasm, and for virulence in murine models of melioidosis. We have recently described the repertoire of effector proteins secreted by the *B. pseudomallei* Bsa system, however the functions of many of these effector proteins remain an enigma. One such protein is BipC, a homolog of the translocator/effector proteins SipC and IpaC from *Salmonella* spp. and *Shigella flexneri* respectively. SipC and IpaC each have separate and distinct roles acting both as translocators, involved in creating a pore in the eukaryotic cell membrane through which effector proteins can transit, and as effectors by interacting with and polymerizing host cell actin. In this study, pull-down assays demonstrate an interaction between BipC and actin. Furthermore, we show that BipC directly interacts with actin, preferentially with actin polymers (F-actin) and has the ability to polymerize actin in a similar manner as that described for SipC. Yet unlike SipC, BipC does not stabilize F-actin filaments, indicating a functionally distinct interaction with actin. Expression of Myc-tagged BipC in HeLa cells induces the formation of pseudopodia similar to that seen for IpaC. This study explores the effector function of BipC and reveals that actin interaction is conserved within the BipC/SipC/IpaC family of translocator/effector proteins.

## Introduction

*Burkholderia pseudomallei* is a Gram-negative saprophyte found in the environment in soil and standing water of Southeast and South Asia, tropical Australia, Western sub-Saharan Africa, and South America. It is the causative agent of melioidosis, a severe disease of both humans and animals (reviewed in Currie, 2015). Melioidosis is an emerging disease predicted to be vastly underreported, with an estimated 165,000 cases of human melioidosis per year resulting in 89,000 deaths (Limmathurotsakul et al., 2016). There is currently no effective vaccine for melioidosis and treatment options are limited due to intrinsic antimicrobial resistance mechanisms (reviewed in Schweizer, 2012). *B. pseudomallei* is a facultative intracellular pathogen capable of invasion of both phagocytic and non-phagocytic cells (Pruksachartvuthi et al., 1990; Jones et al., 1996) which is followed by rapid escape from the endosome (Harley et al., 1998b). Once in the cytosol, the bacterium is capable of polymerizing host cell actin to move both within and between cells by a process known as actin-based motility (Kespichayawattana et al., 2000; Stevens M. P. et al., 2005), as well as fusing together host cell membranes causing the formation multi-nucleated giant cells (Harley et al., 1998a; Kespichayawattana et al., 2000).

One important virulence factor which plays key roles in the *B. pseudomallei* intracellular lifestyle is the Type III Secretion System (T3SS) (reviewed in Sun and Gan, 2010). The T3SS functions as a molecular syringe, allowing delivery of proteins from the bacterial cytosol directly into the cytoplasm of a target host cell, where they subvert host cell functions for the benefit of the bacteria (reviewed in Büttner, 2012). Consisting of around 20 individual structural and regulatory proteins, T3SSs are a key virulence factor in a range of clinically and economically important pathogenic Gram-negative bacteria, such as *Yersinia* spp., *Salmonella* spp., *Shigella flexneri, Escherichia coli*, and *Burkholderia* spp. (reviewed in Coburn et al., 2007).

The genome of *B. pseudomallei* encodes three T3SSs (Winstanley and Hart, 2000; Attree and Attree, 2001; Rainbow et al., 2002). T3SS-1 and T3SS-2 are members of the Hrp family of T3SSs found in bacterial pathogens of plants (Rainbow et al., 2002). T3SS-3, also known as the *Burkholderia* secretion apparatus (Bsa) T3SS, is a member of the Inv-Mxi-Spa family of T3SSs from *Salmonella* spp. (SPI-1) and *S. flexneri* (Attree and Attree, 2001; Stevens et al., 2002; Egan et al., 2014). The Bsa system is required for full virulence in a murine model of melioidosis (Stevens et al., 2004; Burtnick et al., 2008). In *Shigella* and *Salmonella*, T3SSs are involved in cellular invasion followed by either rapid escape into the cytosol (*Shigella*) or alteration of the phagosome to provide a replicative niche (*Salmonella*) (reviewed in Gruenheid and Finlay, 2003). Similarly, the *B. pseudomallei* Bsa T3SS is required for efficient invasion of host cells (Stevens et al., 2003; Muangsombut et al., 2008) and escape from the phagosome (Stevens et al., 2002; Burtnick et al., 2008; Muangsombut et al., 2008).

Recently we characterized the repertoire of proteins secreted by the Bsa T3SS system using quantitative proteomics and identified two novel effector proteins, BprD and BapA, as well as six proteins that had previously been predicted or identified as being secreted in a Bsa-dependent manner (Vander Broek et al., 2015). Yet, in comparison to *Shigella* and *Salmonella*, the functions of many of the effector proteins secreted by the Bsa T3SS remain poorly characterized. One such protein is BipC, which together with BipB likely function as translocator proteins of the Bsa T3SS (Vander Broek et al., 2015). T3SS translocator proteins are responsible for forming a pore in the eukaryotic cell membrane allowing the injection of effector proteins into the cytoplasm, which in the case of the Inv-Mxi-Spa family of T3SSs, requires two distinct proteins (reviewed in Büttner, 2012). BipC shares homology with the SipC and IpaC translocator proteins of *Salmonella* and *Shigella* spp. respectively. Though SipC and IpaC are both bacterial translocators, they also have secondary effector functions, with the ability to directly bind and polymerize host cell actin (Hayward and Koronakis, 1999; Terry et al., 2008). SipC was first shown to directly interact with actin by Hayward and Koronakis (1999). SipC has the ability to polymerize actin as well as bundle F-actin in the absence of host cell proteins (Hayward and Koronakis, 1999; McGhie et al., 2001), and this activity is dependent on the C-terminal half of the 409 amino acid protein (Chang et al., 2005; Myeni and Zhou, 2010). In order to efficiently polymerize actin SipC forms either dimers or multimers (Chang et al., 2007), and another bacterially-encoded actin-binding protein, SipA, stabilizes actin filaments and enhances the polymerization function of SipC (McGhie et al., 2001). The actin polymerization domain of SipC has also been demonstrated to be separate and functionally distinct from the domain required for translocation (Myeni and Zhou, 2010). *Shigella* IpaC also induces actin polymerization when expressed or microinjected into cells (Van Nhieu et al., 1999) and the C-terminus of the protein nucleates actin (Terry et al., 2008). It is clear that IpaC and SipC are multi-functional proteins which play important roles in intracellular life, leading to the hypothesis that BipC may also have distinct roles beyond that of a translocator protein.

In this study, we provide evidence of a direct interaction between BipC and actin. We also demonstrate that BipC has the ability to polymerize actin *in vitro*. HeLa cells expressing N-terminal Myc-tagged BipC were morphologically distinct, displaying multiple pseudopodia suggestive of subversion of the host cell actin cytoskeleton. We clearly show that BipC preferentially binds F-actin, but in contrast to SipC it does not stabilize F-actin under depolymerizing conditions. This study furthers our understanding of the function of BipC in *B. pseudomallei* pathogenesis beyond its role as a Bsa T3SS translocator.

## Methods

### Bacterial strains, plasmids, and culture media

Bacterial strains and plasmids are listed in Table S1. Bacteria were routinely cultured at 37°C on LB agar (Miller) or LB broth (Lennox). Antibiotic selection was performed using ampicillin (Amp) at 100 μg/ml unless otherwise stated. Isopropyl-β-D-thiogalactopyranoside (IPTG) was used at a final concentration of 0.2 mM where appropriate. Eukaryotic tissue culture was performed at 37°C in the presence of 5% CO_2_. HeLa cells were cultured in Dulbecco's Modified Eagle Medium (DMEM, Gibco) supplemented with 10% heat-inactivated fetal calf serum (FCS), 1% L-glutamine, 100 units/ml penicillin, and 100 μg/ml streptomycin.

### Preparation of glutathione S-transferase-fusion proteins

To create N-terminal glutathione S-transferase (GST)-fusion proteins, the full length genes encoding BipC (*B. pseudomallei* 10276) and SipC (*S. enterica* serovar Typhimurium 4/74) were amplified from genomic DNA, cloned into the vector pGEX-4T-1, propagated in *E. coli* XL1 Blue (Agilent) and then confirmed by Sanger sequencing (Source Bioscience). pGEX-BimA_48−384_ (Stevens M. P. et al., 2005) was used as a positive control in relevant assays. pGEX, pGEX-BipC, -SipC, and -BimA_48−384_ were transformed into *E. coli* Rosetta Bl21 (Novagen). An overnight culture of *E. coli* Rosetta BL21 containing pGEX, pGEX-BipC, -SipC, or -BimA_48−384_ was used to inoculate LB containing Amp followed by incubation for 3 h with shaking at 37°C. IPTG was added followed by incubation overnight at 16°C. The bacteria were collected by centrifugation and lyzed with ice-cold lysis buffer [1% Octylthioglucoside, 200 mM NaCl, 1 mM EDTA, 20 mM Tris Cl, 1 mM DTT, 0.1% Triton X-100, 10 μl Lysonase Bioprocessing Reagent (Merck)]. The insoluble fraction was removed by centrifuging at 10,000 RCF. In the case of GST-SipC, the recombinant protein was insoluble after induction. Insoluble GST-SipC was purified from inclusion bodies and refolded using the Novagen protein refolding kit according to the manufacturer's instructions.

For GST, GST-BimA, and GST-BipC, glutathione (GSH)-linked Sepharose beads (GE Healthcare) were added to the bacterial lysate and incubated at 4°C for 1 h with agitation. The beads were collected by centrifugation, washed extensively with PBS followed by Re-suspension in an equal volume of PBS. When necessary, GST-fusion proteins were eluted in elution buffer (50 mM reduced GSH, 50 mM Tris, pH 8.0). The solution was dialyzed three times in PBS for a minimum of 2 h each to remove the detergents and salts. The dialyzed protein was concentrated using a 10 kDa cut-off spin column and protein concentrations determined using a Direct Detect (Merck Millipore).

### Murine splenic lysate pull-downs

Murine spleens^*^ were homogenized in 1x polymerization buffer with protease inhibitors (10 mM Tris—pH 7.5, 1 mM ATP, 2 mM MgCl_2_, 50 mM KCl, 0.5 mM EGTA, 1 mM PMSF, 1 μg/ml pepstatin A, 10 μg/ml leupeptin, 1 μg/ml aprotinin). Lysates were clarified by ultracentrifugation at 100,000 RCF. To perform pull downs, an aliquot of murine splenic lysate was diluted 1:3 with 1x polymerization buffer and CaCl_2_ was added to a final concentration of 100 μM. The GST or GST-fusion proteins of interest (-BimA_48−384_, -BipC, and -SipC) bound to GSH-linked Sepharose beads were added to the lysate and incubated for 1 h at room temperature with agitation. The Sepharose beads were then washed five times with ice-cold PBS. Samples were subjected to SDS-PAGE and visualized by Coomassie blue staining (Thermo Fisher). For immunoblotting, proteins were transferred to PVDF membranes and visualized using goat α-actin primary antibody (Santa Cruz Biotechnology) followed by rabbit α-goat HRP-linked secondary antibody (Invitrogen). The antibodies were detected using ECL (Thermo Fisher) followed by film exposure.

^*^Tissues were obtained from wild-type mice in accordance with the UK Animals (Scientific Procedures) Act 1986, following local ethical review of protocols.

### Rhodamine-actin binding assay

Confocal microscopy was used to investigate the ability of GST-fusion proteins to directly bind actin as described in (Stevens J. M. et al., 2005; Stevens M. P. et al., 2005). To the center of a glass microscope slide, 10 μl of 1x polymerization buffer or PBS containing 1 μM rhodamine labeled actin was added. To this, 1 μl of the GST-fusion proteins of interest (GST, GST-BimA_48−384_, or GST-BipC) bound to GSH-linked Sepharose beads was added. The slide was immediately visualized using a Zeiss LSM 710 Scanning Laser Confocal Microscope using an excitation of 535 nm and an emission of 585 nm, and data collected using Zen 2011 software (Zeiss). Images were further processed using Image J (https://imagej.nih.gov/ij/).

### Actin sedimentation assay

Mg-actin was prepared by incubating 4 μg/μl rhodamine-labeled actin (Cytoskeleton) in the presence of 50 μM MgCl_2_, and 125 μM EGTA for 10 min at room temperature. To the mixture, 10x polymerization buffer was added to a final concentration of 1x and incubated for 2 h at room temperature to polymerize the G-actin into F-actin. Sedimentation reactions were prepared in G-Mg buffer (5 mM Tris-HCl pH8, 0.2 mM MgCl_2_, 0.2 mM ATP, 0.5 mM DTT) by adding 2.3 μM of F-actin and 1 μM of GST-fusion protein. The reaction was incubated at room temperature for 30 min in the dark before being centrifuged at 100,000 RCF for 1 h at 20°C to sediment the F-actin. The soluble fraction (monomeric G-actin fraction) was separated from the pellet (filamentous F-actin) and the fractions were re-suspended in 2x Laemmli buffer, separated by SDS-PAGE and visualized using Silver staining (Pierce Scientific) according to the manufacturer's protocol. Densitometry was performed using Image Studio Lite software (LI-COR Biosciences).

### Pyrene actin polymerization assay

A pyrene actin polymerization assay was performed essentially as previously described (Stevens et al., 2003). Samples were excited with a wavelength of 365 nm and emission was collected at a wavelength of 407 nm. Data was collected every 30 s for up to 1 h using a FLUOstar Omega plate reader (BMG Labtech). Monomeric actin was used in polymerization assays at a final concentration of 2–3 μM and GST or GST-fusion proteins were used at a final concentration of 1 μM. Each well contained polymerization buffer at a final concentration of 1x. The assay was performed in three independent replicates with three technical measurements per replicate. Data were plotted in Excel. The rates of polymerization were calculated as the rise in fluorescence units per second during the linear phase of polymerization, between 200 and 800 s. The mean rates of polymerization for each protein were analyzed by pairwise Student *t*-test using R software (https://www.r-project.org/) and *P*-values of ≤ 0.05 were taken as significant.

### Actin depolymerization assay

To assess the ability of BipC to stabilize actin filaments, an actin depolymerization assay was performed essentially as previously described (McGhie et al., 2001). Mg-actin was prepared by incubating 1 mg/ml pyrene-labeled actin with 125 μM EGTA and 50 μM MgCl_2_ for 10 min at room temperature. The Mg-actin was then polymerized by adding 10x polymerization buffer to a final concentration of 1x, followed by incubation at room temperature in the dark for 2–4 h. The depolymerization assay was set up in a 96 well black opaque plate with each well containing 2.3 μM F-actin, plus or minus 2.5 μM of the GST-fusion proteins. Data was collected every 30 s for up to 1 h using a FLUOstar Omega plate reader (BMG Labtech), with samples excited at 365 nm and emission data collected at 407 nm. The assay was performed in three independent replicates with two measurements per replicate. Data was plotted in Excel. Relative fluorescence intensity was calculated by dividing all of the sample measurements by its value at time = 0 s. The rates of depolymerization were calculated as the decrease in relative fluorescence units per second during the linear phase of depolymerization, between 100 and 600 s. The mean rates of depolymerization for each protein were analyzed by pairwise Student *t*-test using R software (https://www.r-project.org/) and *P*-values of ≤ 0.05 were taken as significant.

### HeLa cell transfections and immunofluorescence microscopy

In order to investigate the effects of ectopically expressed BipC in eukaryotic cells, HeLa cells were transfected with the constitutive eukaryotic expression vector pRK5-myc-BipC, which encodes BipC with an in-frame N-terminal c-Myc tag. The plasmid pEGFP (Clontech) which encodes enhanced green fluorescent protein (EGFP) was used as a control. HeLa cell transfections were performed using Lipofectamine 2000 (Invitrogen) according to the manufacturers protocol, using 4.0 μg of plasmid and 10 μl of Lipofectamine 2000 per well. The transfected HeLa cells were incubated for 48 h before being fixed in 4% paraformaldehyde in PBS. The cells were washed twice with PBS and permeabilized using PBS containing 0.5% Triton X-100 for 15 min. Cells were washed twice with PBS and then blocked for 30 min with blocking buffer (PBS containing 0.5% bovine serum albumin w/v, 0.02% NaN_3_ w/v). In order to detect the Myc-BipC protein, cover slips were incubated with rabbit α-c-Myc primary antibody (0.5 μg/ml, Santa Cruz) at 37°C for 1 h. The cover slips were washed three times with PBS followed by incubation with goat α-rabbit^488^ secondary antibody (10 μg/ml, Molecular Probes) for 1 h. The cover slips were washed three times with PBS and incubated with phalloidin^568^ (10 μg/ml, Invitrogen) for 15 min. The cover slips were washed six times with PBS, mounted and visualized using a Zeiss LSM 710 Scanning Laser Confocal Microscope using Zen 2011 software (Zeiss). Images were processed using Image J software (https://imagej.nih.gov/ij/).

## Results

### BipC directly binds cellular actin

**Figure 1:**
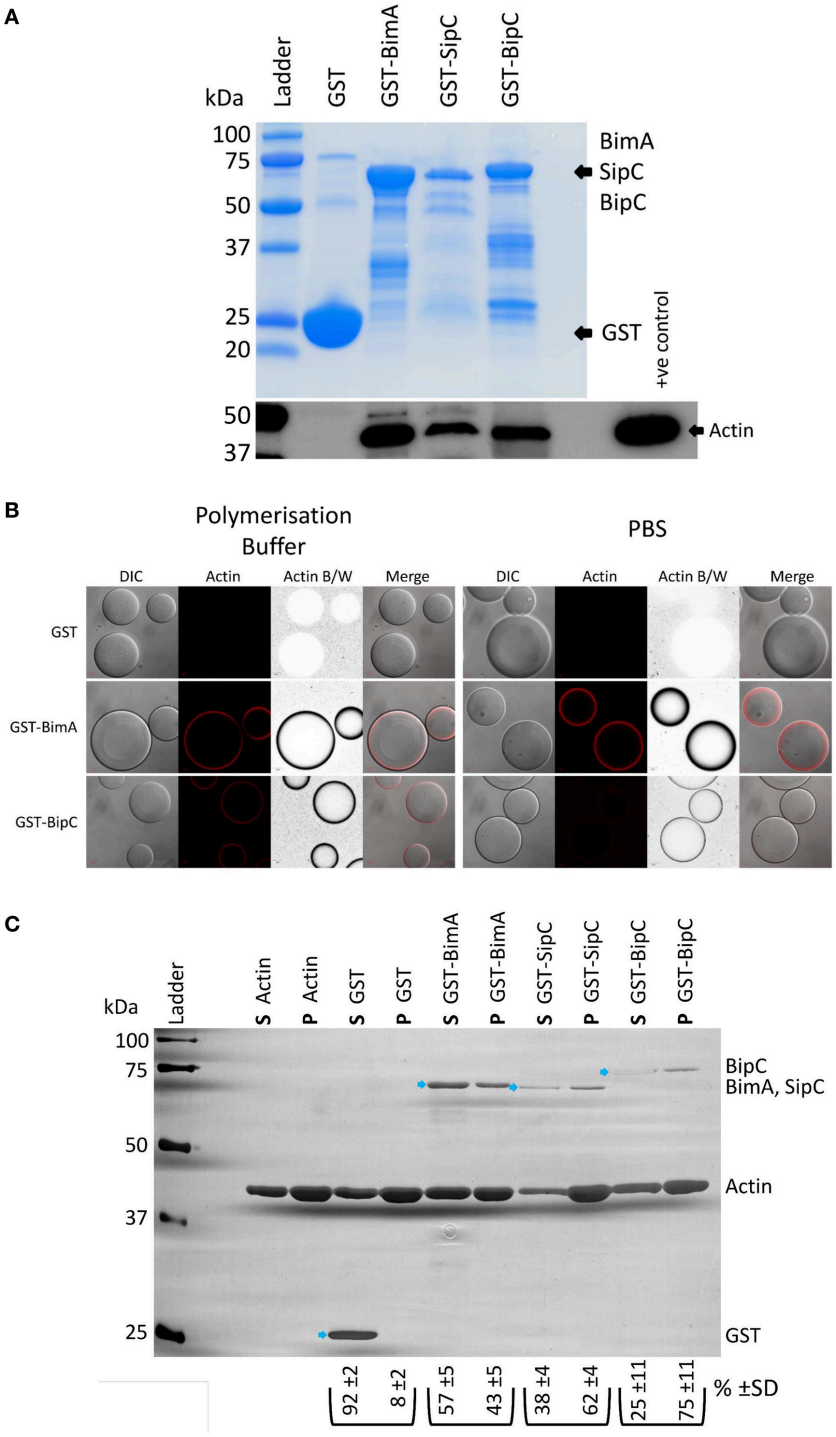
*B. pseudomallei* BipC interacts with cellular actin. **(A)** GST-BipC interacts with actin in murine splenic lysates. GST, GST-BimA_48−384_, GST-SipC, and GST-BipC bound to GSH-linked Sepharose beads were incubated with murine splenic lysates in polymerization buffer. The Sepharose beads were washed and the bound proteins were denatured in Laemmli buffer. The Coomassie stained gel shows the relative quantities of input proteins used in the pull-down assay. Equal volumes (5 μl) of each pulldown sample (representing a half of the total samples) were analyzed by SDS-PAGE and Western blot. The α-actin Western blot indicates actin binding to the fusion proteins. **(B)** GST-BipC directly interacts with actin in the absence of other cellular proteins. GSH-Sepharose beads coated with either GST, GST-BimA_48−384_,or GST-BipC were mixed with rhodamine-labeled actin suspended either in polymerization buffer or PBS and immediately imaged using a confocal microscope. The formation of a red “halo” around the bead indicates binding of actin to the bead surface. These are also shown as grayscale images for clarity. DIC/phase contrast images of the beads are also shown. Scale bar = 20 μm. **(**C**)** GST-BipC preferentially binds F-actin. Actin was allowed to polymerize at room temperature for 2 h before being mixed with GST, GST-BimA_48−384_, GST-SipC, or GST-BipC. The mixtures were submitted to ultra-centrifugation to separate the monomeric actin (supernatant) and the filamentous actin (pellet). Proteins in the supernatant (**S**) and pellet (**P**) fractions were separated by SDS-PAGE and visualized by silver staining. The GST-fusion proteins are indicated by the blue arrows. The average percentage and standard deviation over the three replicates of each GST-fusion protein distributed in the supernatant or pellet (as determined by densitometry) is shown below the corresponding lane in the image (shown as **%**).

BipC (annotated as BPSS1531 in the reference K96243 genome) is encoded within the Bsa T3SS locus and is very highly conserved in pathogenic *B. pseudomallei* and *B. mallei* (100% identity), and in the closely related avirulent *B. thailandensis*, though to a lesser extent (86% identity). The *B. thailandensis* BipC protein contains numerous single amino acid differences as well as two minor deletions in comparison to the BipC proteins of the other two species (Figure S1). BipC shares homology with SipC (58% coverage and 24% identity at the amino acid level) and IpaC (58% coverage, 22% identity) from *Salmonella* spp. and *S. flexneri*, respectively (Figure S2). We previously identified BipC in the secretome of *B. pseudomallei* using a quantitative proteomics approach (Vander Broek et al., 2015). Due to the similarity in sequence between BipC and SipC/IpaC, we hypothesized that BipC would be an actin-binding protein and may possess the ability to bundle F-actin. In the following experiments, we have directly compared the activities of BipC with *Salmonella* SipC or the *B. pseudomallei* BimA protein as positive controls. We have previously characterized BimA, which is required for actin-based motility of *B. pseudomallei*, as an actin monomer binding and actin polymerizing factor (Stevens M. P. et al., 2005; Sitthidet et al., 2011). Both SipC and IpaC interact with and nucleate actin polymerization *in vitro* (Hayward and Koronakis, 1999; Terry et al., 2008), facilitating bacterial invasion of host cells (Mounier et al., 2009; Myeni and Zhou, 2010). In order to investigate whether BipC associates with cellular actin, pull-down experiments were performed using a GST-BipC fusion protein and murine splenic lysates under actin polymerizing conditions. In a similar manner to the control SipC and BimA proteins, Figure 1A shows that BipC can interact with actin in a murine splenic lysate. Because pull-downs may identify interactions that are indirect through large protein complexes (reviewed in Kool et al., 2011), further work was required to determine if BipC has the ability to bind actin directly. To test this, GSH-linked Sepharose beads coated with GST fusion proteins were incubated with rhodamine-labeled actin, under either polymerizing or non-polymerizing conditions (PBS), followed by immediate visualization using a confocal microscope. A protein that is able to directly bind actin creates a high concentration of the rhodamine-labeled actin at the surface of the Sepharose bead, which appears as a red “halo” or ring around the clear bead in microscope images. The GST-BimA_48−384_ coated beads directly bound actin in both polymerization buffer and PBS as indicated by a distinct red halo (Figure 1B), and in agreement with previous studies by our laboratory (Stevens J. M. et al., 2005; Stevens M. P. et al., 2005; Sitthidet et al., 2011). The GST-BipC coated beads also directly bound actin in both polymerization buffer and PBS, but appeared to bind actin more efficiently under polymerizing conditions (Figure 1B). At the time of submission of this manuscript for peer review we believed this to be the first evidence that BipC is an actin-binding protein. However, we have since become aware of a similar study by Kang et al. (2016), which also demonstrates that *B. pseudomallei* BipC is an actin-binding protein.

As BipC appeared to bind actin more efficiently under polymerizing conditions, we were interested in testing whether it preferentially binds to F-actin or G-actin using a quantitative sedimentation assay as we have previously described (Stevens M. P. et al., 2005). Purified GST and GST-fusion proteins (GST-BipC, GST-SipC, and GST-BimA_48−384_) were pre-incubated with polymerized actin (F-actin). Ultra-centrifugation was then used to sediment the F-actin along with any interacting GST fusion protein. Under these conditions the monomeric G-actin, along with any G-actin interacting proteins or with non-interacting proteins (such as GST), remain in the supernatant. The supernatants and pellets were then subjected to SDS-PAGE and visualized by silver staining. Densitometry was used to quantify the levels of protein present in each sample. Three independent replicates of the assay were performed to ensure reproducibility. As a control, the assay was also performed in the absence of actin to demonstrate that the GST proteins would not sediment on their own (Figure S3). As expected the GST protein was primarily present in the supernatant fraction as it does not possess actin binding activity (Figure 1C). GST-BimA_48−384_ was also most abundant in the supernatant fraction due to its intrinsic G-actin binding activity (Figure 1C), and in concordance with our previous published observation (Stevens M. P. et al., 2005). GST-SipC associated predominantly with the pellet fraction due to its preferential binding to F-actin, which is in agreement with data published by Hayward and Koronakis (1999) (Figure 1C). Similarly, GST-BipC also co-sedimented with the F-actin pellet in this assay, indicating that it too preferentially binds F-actin under these conditions (Figure 1C).

We have recently investigated the interaction of *B. pseudomallei* BimA with cellular proteins using a yeast two-hybrid assay (Jitprasutwit et al., 2016). Whilst we have been able to confirm that BimA is an actin-interacting protein in this assay (Jitprasutwit et al., 2016), we were unable to detect a direct interaction between BipC and actin by yeast two-hybrid assay (Figure S4). In our assay, the BipC protein was fused to the GAL4 DNA-binding domain and actin was fused to the GAL4 activation domain. It is possible that the fusion of BipC to the GAL4 DNA-binding domain affected the tertiary structure of the protein and disrupted the interaction. It is also a possibility that the actin-GAL4 fusion protein is unable to polymerize into F-actin, which we have demonstrated that BipC predominantly associates with (Figure 1C). In support of this hypothesis, we have been unable to find examples of the use of the yeast two-hybrid system to dissect interactions of F-actin binding proteins with actin in the literature.

### BipC polymerizes actin *In vitro*

**Figure 2:**
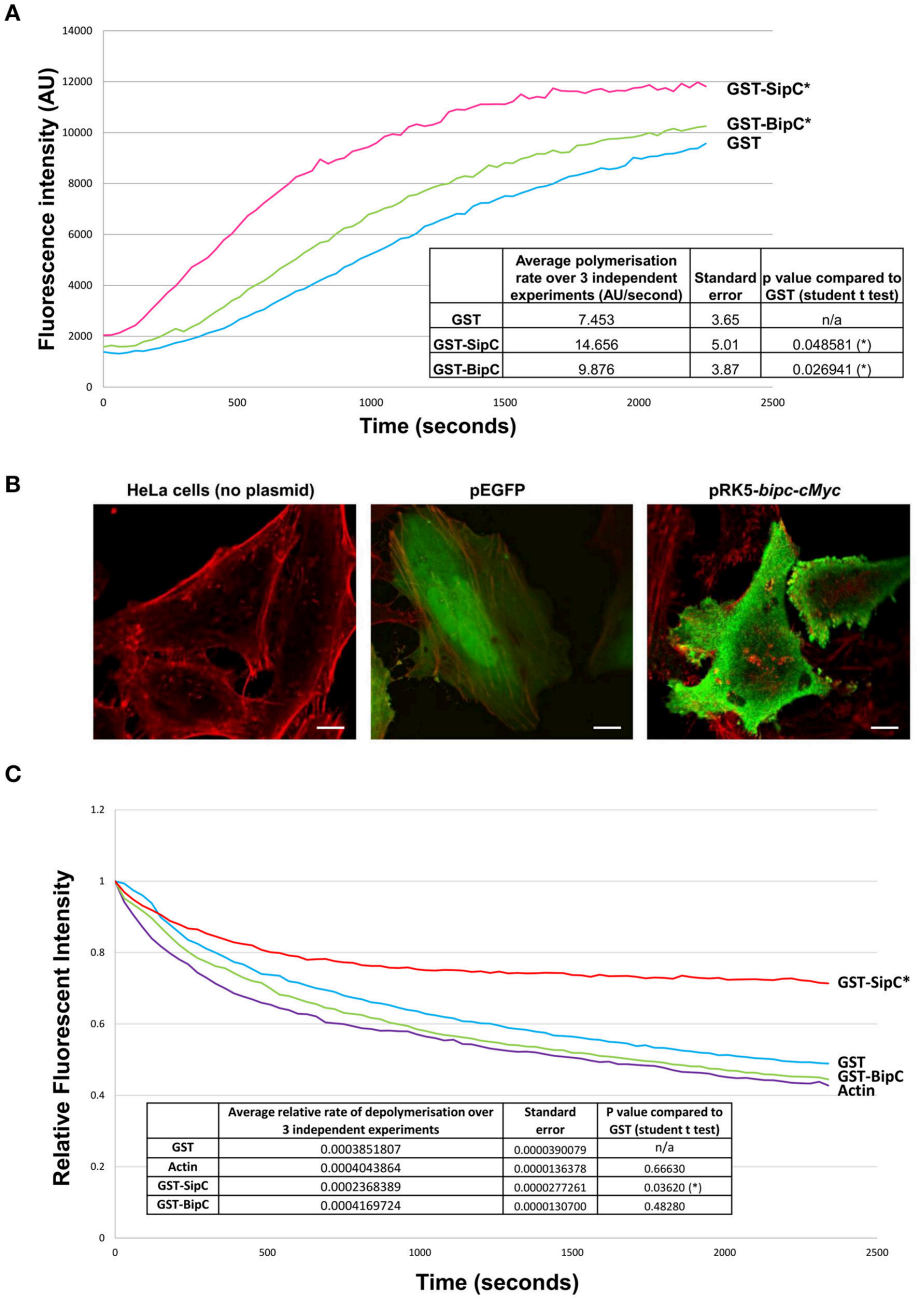
BipC demonstrates actin polymerization, but not F-actin stabilizing activities. **(A)** BipC stimulates actin polymerization *in vitro*. GST, GST-SipC, and GST-BipC were mixed with monomeric pyrene-actin in actin polymerization buffer. The polymerization assay was monitored every 30 s for 40 min with an excitation wavelength of 365 nm and an emission wavelength of 407 nm. The rate of polymerization was calculated between 200 and 800 s. The graph shows one representative experiment, while the table insert shows data accumulated from three independent experiments, each consisting of three technical replicates. **(B)** Ectopic expression of Myc-tagged BipC alters cell morphology. HeLa cells were transfected with pRK5-Myc-BipC and fixed in paraformaldehyde 48 h post-transfection. Following permeabilization, cells were probed using phalloidin and rabbit α-c-Myc antibodies, and imaged using a confocal microscope. HeLa cells transfected with pEGFP were also imaged. In the merged representative images, the actin cytoskeleton stained with phalloidin appears red, and Myc-BipC or pEGFP appear green. Pseudopodia-like structures are indicated by the white arrows. **(C)** BipC lacks F-actin stabilization activity. GST, GST-BipC, and GST-SipC were incubated with pyrene labeled F-actin. Fluorescence was measured for 40 min in 30 s intervals with an excitation wavelength of 365 nm and an emission wavelength of 407 nm. The rate of depolymerization was calculated between 100 and 600 s. The graph shows one representative experiment. The table insert shows the accumulated data from three independent experiments, each consisting of two technical replicates.

Previous work has demonstrated that *Salmonella* SipC not only has the ability to bind and bundle F-actin, but also to polymerize actin (Hayward and Koronakis, 1999). To determine if BipC is also able to polymerize actin, a polymerization assay was performed with pyrene-labeled actin, which emits fluorescence over time as monomers assemble into actin filaments (Stevens M. P. et al., 2005). GST, GST-SipC, and GST-BipC were incubated with pyrene actin in polymerization buffer and their rates of actin polymerization compared by measuring fluorescence intensity over time. SipC showed a statistically significant increased rate of polymerization (average rate of increase in fluorescence intensity = 14.66 ± 5.01 AU/s) when compared to the GST control which mimics the rate of intrinsic actin polymerization in the absence of a polymerizing factor (average rate of increase in fluorescence intensity = 7.45 ± 3.65 AU/s) (Figure 2A). BipC also showed a statistically significant increase in the rate of actin polymerization (average rate of increase in fluorescence intensity = 9.88 ± 3.87 AU/s) when compared to the GST control indicating that this protein has an intrinsic actin polymerizing activity (Figure 2A).

With the establishment that BipC binds and polymerizes actin *in vitro*, we wanted to investigate what effect BipC would have when expressed ectopically in eukaryotic host cells. Full length BipC was transiently expressed with an in-frame N-terminal c-Myc tag in HeLa cells. At 48 h post-transfection, the cells were fixed and permeabilized, probed using phalloidin (to label F-actin) and rabbit α-Myc antibodies to label BipC, and imaged using a confocal microscope. In cells expressing BipC, the BipC protein localized around the periphery of the cells (Figure 2B). The cells expressing BipC were morphologically distinct from untransfected cells, displaying multiple pseudopodia-like structures, indicating that BipC modulates actin dynamics within host cells (Figure 2B).

### BipC lacks the ability to stabilize F-actin

In addition to stimulating the polymerization of actin *in vitro, Salmonella* SipC also stabilizes F-actin polymers, preventing their dissociation in solution. The actin polymerizing and F-actin bundling activities of SipC are enhanced by another T3SS effector protein, SipA (McGhie et al., 2001). Interestingly, there is no direct homolog of SipA encoded by the *B. pseudomallei* genome. To investigate whether BipC has the ability to stabilize F-actin polymers in its own right, an actin depolymerization assay was performed. The inverse of the polymerization assay, the depolymerization assay measures a reduction in the rate of relative fluorescence over time as pyrene-F-actin filaments disassemble into actin monomers (G-actin). GST, GST-SipC, or GST-BipC were incubated with pre-polymerized pyrene-actin under depolymerizing conditions and fluorescence intensity was measured over time. As expected, the GST negative control showed similar levels of depolymerization to that of the actin-only control since it lacks any actin-binding or F-actin stabilizing activities (Figure 2C). Under the same assay conditions F-actin incubated with SipC was significantly stabilized and showed significantly slower rates of depolymerisation, as previously demonstrated by McGhie et al. (2001). Surprisingly we did not need to include recombinant SipA in our assay, although addition of SipA may well have further enhanced the F-actin stabilizing activity of SipC. In contrast the GST-BipC protein showed no significant F-actin stabilizing activity in this assay, demonstrating that this protein, unlike its homolog SipC, does not have intrinsic F-actin bundling activity (Figure 2C). Since submission of this manuscript for peer review, a study by Kang et al. (2016) suggested that a His-tagged BipC protein exhibited F-actin stabilizing activity in a similar assay. However, it is notable that much higher concentrations of BipC protein were required in these experiments than used in our study, or that of McGhie et al. (2001).

## Discussion

The *B. pseudomallei* BipC protein is homologous to the *Salmonella* SipC and *Shigella* IpaC T3SS proteins, and is secreted by the Bsa T3SS (Stevens et al., 2002; Vander Broek et al., 2015). A predicted translocator protein, BipC is highly conserved in all *B. pseudomallei, B. mallei* and *B. thailandensis* strains sequenced to date. The cognate translocator protein responsible for pore formation in the eukaryotic cell membrane is known as BipB. Both BipB and BipC are potent B-cell antigens (Felgner et al., 2009), however, BipC recombinant proteins failed to demonstrate efficacy as subunit vaccines in murine models of melioidosis (Druar et al., 2008). A recent study demonstrated that a *bipC* insertion mutant was attenuated in a BALB/c mouse model of melioidosis, showed significant defects in host cell adhesion, invasion, phagosome escape and intracellular replication (Kang et al., 2015). Similar findings have been reported upon ablation of the *bipB* gene (Suparak et al., 2005). While the Kang study (2015) shows a clear role of BipC in virulence and the intracellular lifestyle of the bacterium, it does not separate BipC's role as a translocator (necessary for a functional T3SS) from additional roles it may have as an effector protein. Here we have sought to characterize the effector function of BipC in relevant biochemical assays utilizing our knowledge of the effector functions of the homologous *Salmonella* SipC and *Shigella* IpaC proteins.

Both SipC and IpaC bind and nucleate actin (Hayward and Koronakis, 1999; Terry et al., 2008), facilitating bacterial invasion of host cells (Mounier et al., 2009; Myeni and Zhou, 2010). The actin nucleation domain of IpaC has been identified as the C-terminal 20 amino acids (Terry et al., 2008), an area relatively more highly conserved in BipC (Figure S2). Here we have demonstrated that GST-BipC has the ability to bind actin directly, in the absence of other bacterial or cellular proteins. We acknowledge that whilst our study was in review, Kang et al. (2016) published similar observations to those presented in our manuscript. However, upon closer inspection there is only a small amount of “overlap” with our data regarding the function of BipC as an actin binding and polymerizing factor. Kang et al. (2016) demonstrated that BipC interacts with both monomeric and filamentous actin using a basic pulldown assay, however there is no quantitation or inclusion of relevant control proteins in their assay. What we add to this finding is that BipC preferentially binds F-actin filaments, behaving in a manner more similar to SipC than BimA (Stevens M. P. et al., 2005; Myeni and Zhou, 2010). We believe we have been significantly more thorough in our investigation of the BipC: actin interaction and the ability of BipC to polymerise actin *in vitro*, utilizing several different approaches and applying quantification methods where appropriate. We also believe that we have included relevant and pertinent controls in all of the assays presented in this paper. In addition, a noteworthy finding in our study was the inability of BipC to stabilize F-actin, suggesting that its role in actin dynamics differs from that of SipC. This may not be surprising as the ability of SipC to bundle F-actin has been attributed to amino acids 1–120 (Hayward and Koronakis, 1999) and 221–260 (Myeni and Zhou, 2010), regions which are not well-conserved in BipC (Figure S2).

Beyond its ability to directly bind actin, it was also demonstrated that BipC polymerizes actin *in vitro* in the absence of any other bacterial or cellular co-factors. In our hands, BipC-mediated actin polymerization appeared to be less potent than that displayed by SipC under the same assay conditions. Further evidence in support of a role for BipC in polymerizing actin was presented following ectopic expression of the protein in eukaryotic host cells. The Myc-tagged BipC protein localized to the cell membrane with F-actin and pseudopodia-like structures. A similar phenotype has been described upon introduction of recombinant IpaC into permeabilized Swiss 3T3 cells (Van Nhieu et al., 1999), as well as in NIH3T3 cells ectopically expressing SipC (Cain et al., 2004).

It has been hypothesized that the SipC/IpaC family of translocator/effector proteins may play a pivotal role in determining the intracellular niche of the bacteria. A *Salmonella sipC* mutant can be complemented by both *sipC* or *ipaC* expression *in trans*, but a *Shigella ipaC* mutant can only be complemented by *ipaC* (Osiecki et al., 2001). The authors suggest the different functions may parallel the different intracellular lifestyles of the pathogens, with *Salmonella* residing within the vacuole and *Shigella* rapidly escaping into the cytosol (Osiecki et al., 2001). Indeed, this is supported by a recent study in which *Salmonella* expressing *ipaC* was shown to be capable of vacuole escape (Du et al., 2016). Because of closer parallels between the intracellular lifestyles of *B. pseudomallei* and *Shigella*, we would predict that BipC would function in a manner more similar to IpaC than SipC, mediating the exit of the bacterium from the endocytic compartment into the host cell cytosol.

The differences between *Salmonella* and *Shigella* also extend to other T3S actin modulating proteins. It has been shown that the actin polymerization activity of SipC can be enhanced by *Salmonella* T3SS effector protein SipA, but this activity cannot be functionally complemented by the homologous *Shigella* T3SS effector, IpaA (McGhie et al., 2001). IpaA has instead been shown to bind cellular vinculin altering its barbed end capping activity, causing depolymerisation of F-actin at the site of bacterial entry (Van Nhieu et al., 1997; Bourdet-Sicard et al., 1999; Ramarao et al., 2007; Park et al., 2011). It is possible that other *B. pseudomallei* T3SS effector proteins, that have yet to be identified, may work synergistically with BipC to enhance actin polymerization or, similar to IpaA, may affect cellular actin dynamics in some other way.

Further work is now warranted to explore the mechanisms by which BipC binds and nucleates actin, as well the role this may play in *B. pseudomallei* pathogenesis. It is tempting to speculate that the effector function of BipC is involved in the invasion of host cells and/or the escape from the phagosome. Nonetheless, it is clear that a better understanding of *B. pseudomallei* pathogenesis is required in order to facilitate the development of improved control measures and alternative therapies for melioidosis in the future.

## Author contributions

CV and JS designed the experiments. CV, NZ, and JS conducted the experiments and analyzed the data. CV and JS prepared the manuscript.

### Conflict of interest statement

The authors declare that the research was conducted in the absence of any commercial or financial relationships that could be construed as a potential conflict of interest.
