# Supplementary material for: BipC, a Predicted Burkholderia pseudomallei Type 3 Secretion System Translocator Protein with Actin Binding Activity
Source: Front Cell Infect Microbiol. 2017 Jul 19;7:333. doi: 10.3389/fcimb.2017.00333 (PMC5515863; doi:10.3389/fcimb.2017.00333)
Supplement: Supplementary file 1 [file Table1.DOCX]

**BipC, a *Burkholderia pseudomallei* Type 3 Secretion System translocator protein with actin binding activity**

**Charles W. Vander Broek, Nurhamimah Zainal Abidin, Joanne M. Stevens***

The Roslin Institute and Royal (Dick) School of Veterinary Studies, University of Edinburgh, Easter Bush, Midlothian, EH25 9RG, Scotland, UK.

*Corresponding Author

Email: jo.stevens@roslin.ed.ac.uk

**Supplemental methods, figures, tables and references**

**Supplemental Methods**

**Yeast Two-Hybrid Assay**

Yeast two-hybrid was performed using the Matchmaker GAL4 Two-Hybrid system 3 (Clontech), essentially as described in the manufacturer’s instructions. The full coding sequence of BipC was cloned into pGBKT7 (GBKT7-BipC) and the full coding sequence of β-actin was cloned into pGADT7 (pGADT7-Actin). Following verification by sequencing, pGBKT7-BipC and pGADT7-Actin were co-transformed into *S. cerevisiae* strain AH109 using lithium acetate and were cultured on SD media lacking leucine and tryptophan (DDO: Double Drop Out media, Clontech) to create the strain *S. cerevisiae* AH109 pGBKT7-BipC pGADT7-Actin. To test for activation of the reporter genes the *S. cerevisiae* AH109 pGBKT7-BipC pGADT7-Actin was streaked onto SD agar plates lacking leucine, tryptophan, adenine and histidine (QDO: Quadruple Drop Out, Clontech). Plates were incubated for 5 days at 30^o^C and monitored regularly for growth of colonies. Control strains transformed with positive and negative control vector combinations (pGADT7-T antigen/ pGBKT7-p53 and pGADT7-T antigen/ pGADT7-Lamin C, respectively) were included in the assay.

**Table S1: Strains and Plasmids used in this study.**

| **Strain or Plasmid** | **Characteristics** | **Source** |
| --- | --- | --- |
| **Strains** |  |  |
| *E. coli* XL1 Blue | *recA1 endA1 gyrA96 thi-1 hsdR17 supE44 relA1 lac* [F´ *proAB lacI^q^ Z∆M15* Tn*10* (Tet^r^ )] | Agilent |
| *E. coli* Rosetta BL21 | F^-^ *ompT hsdS*B(rB^-^ mB^-^) *gal dcm* (DE3) pRARE (Cam^R^) | Novagen |
| **Burkholderia pseudomallei* 10276 | Isolated from a clinical case of melioidosis. | Maegraith and Leithead 1964 |
| *Salmonella enterica* serovar Typhimurium 4/74 | Encodes full length SipC. | Paulin et al. 2007 |
|  |  |  |
| **Plasmids** |  |  |
| pGEX-4T-1 | Bacterial expression vector, tac promoter, N-terminal GST tag, Amp^R^ | GE Healthcare |
| pGEX-4T-1-BimA_48-384_ | pGEX-4T-1 containing the coding sequence for amino acids 48-384 of BimA from *B. pseudomallei* 10276 | Stevens et al. 2005 |
| pGEX-4T-1-SipC | pGEX-4T-1 containing the full coding sequence of SipC from *S. enterica* Typhimurium 4/74 | This study |
| pGEX-4T-1-BipC | pGEX-4T-1 containing the full coding sequence of BipC from *B. pseudomallei* 10276 | This study |
| pEGFP | Mammalian expression vector expressing EGFP | Clontech |
| pRK5-myc | Mammalian expression vector, CMV promoter, C-terminal Myc tag, Amp^R^ | Clontech |
| pRK5-myc-BipC | pRK5-Myc containing the full coding sequence of BipC from *B. pseudomallei* 10276 | This study |

***** The *B. pseudomallei* strain 10276 was used in these experiments only to provide the genomic DNA required for the cloning of the *bipC* gene into the expression vectors described in the table. This work was undertaken in the United Kingdom through funding obtained from UK sources and in strict accordance with UK Health and Safety Executive (HSE) guidance. Our work in relation to genetic modification has been extensively reviewed both by our own University of Edinburgh Biosafety Unit, the HSE and by the UK Counter Terrorism and Security Agency. No genetically modified B. pseudomallei strains were produced in this study, therefore our work does not constitute a US Government Dual Use of Research Concern.

**Figure S1**


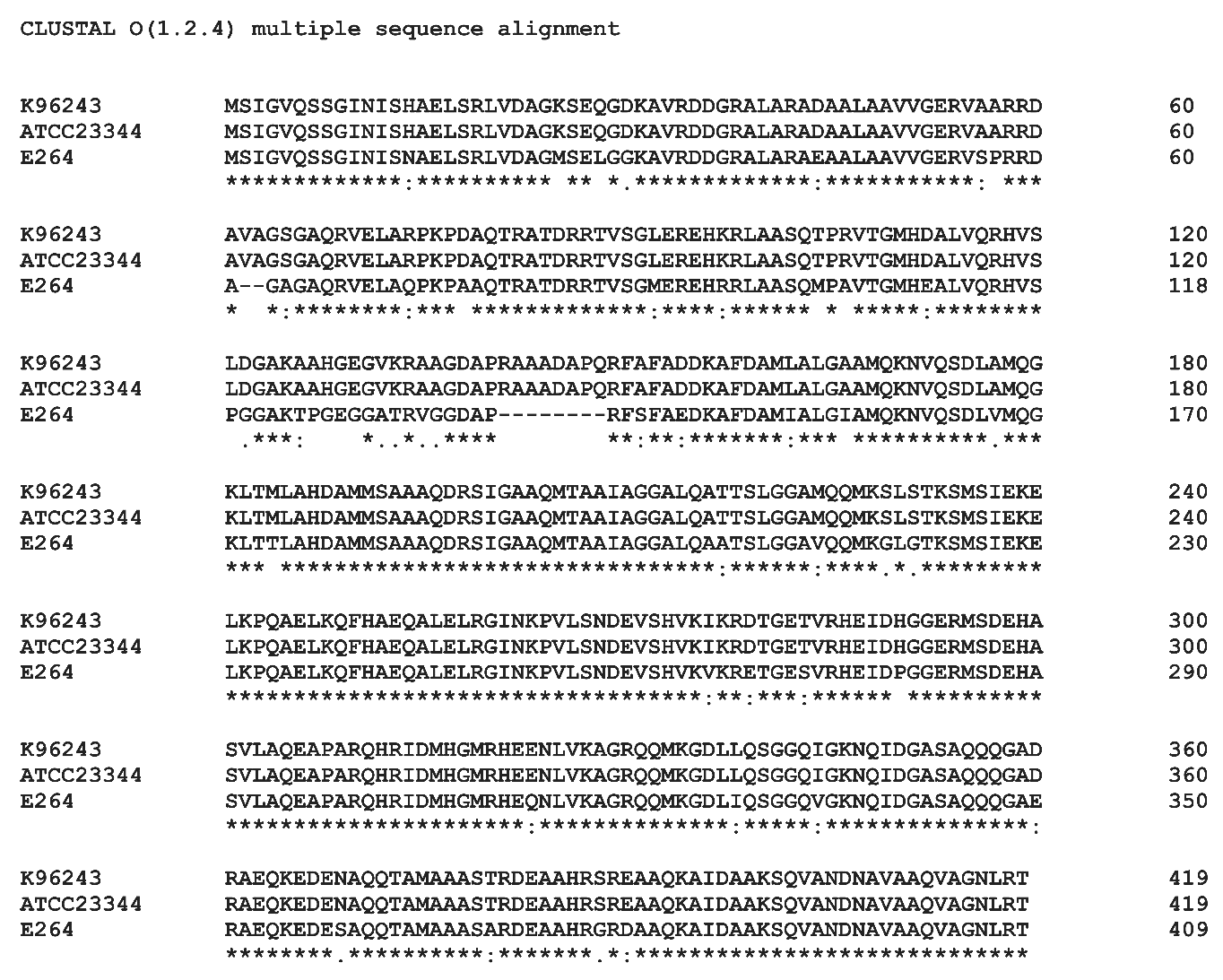


**Figure S1: Amino acid alignment of prototypic BipC proteins from *B. pseudomallei*, *B. mallei* and *B. thailandensis*.**

The alignment was produced using Clustal Omega (Sievers et al. 2011). In the alignment, (*) indicates conserved residues, (:) indicates highly similar amino acids, and (.) indicates weakly similar amino acids. The bacterial strains used for the alignment are *B. pseudomallei* K96243, *B. mallei* ATCC23344, and *B. thailandensis* E264, with Uniprot accession numbers Q63K35, Q62B08, and Q2T710, respectively.

**Figure S2**


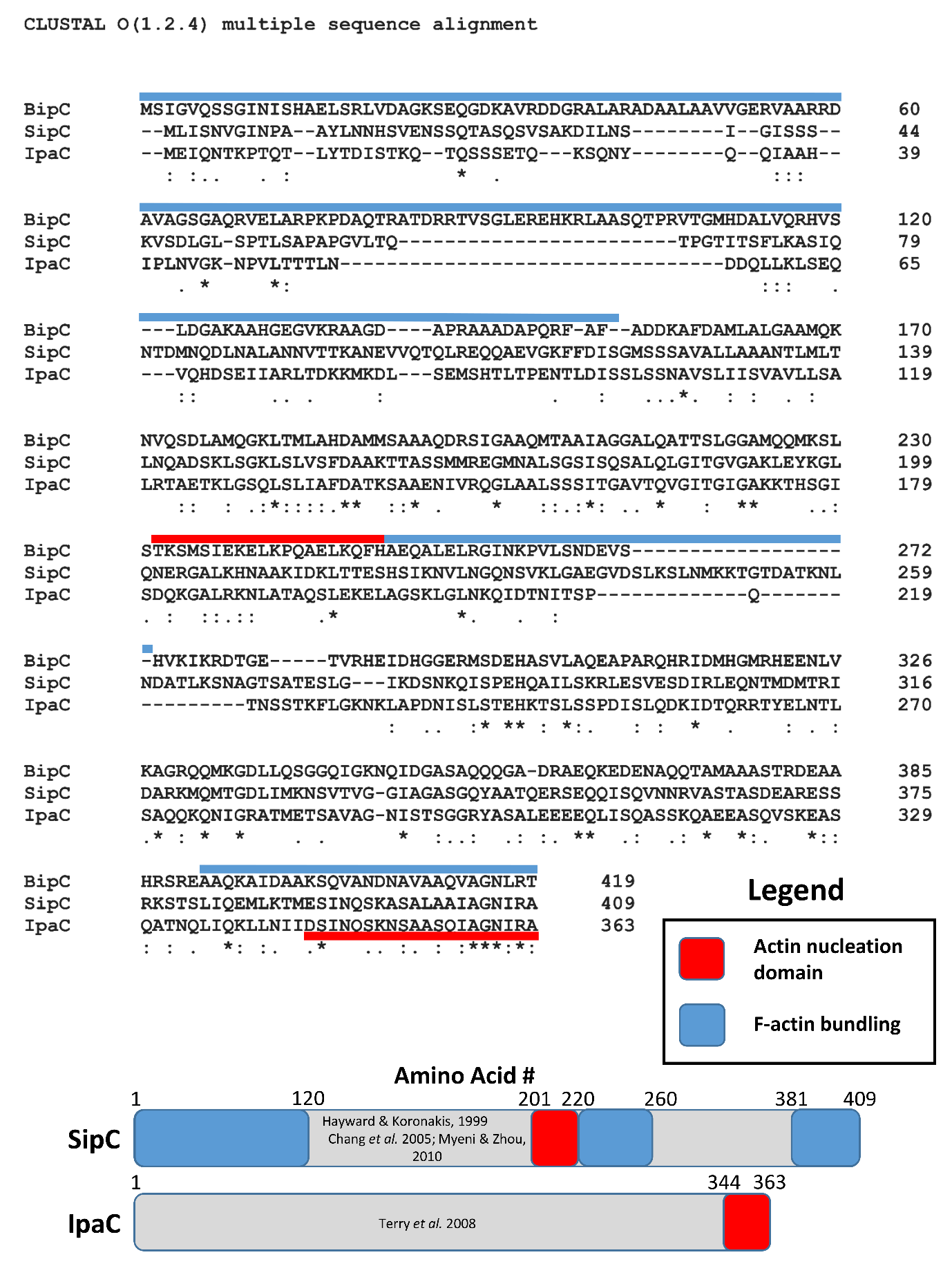


**Figure S2: Domain map and amino acid alignment of *Salmonella* *enteritidis* SipC and *Shigella* *flexneri* IpaC with BipC.**

The alignment was produced using Clustal Omega (Sievers et al. 2011). In the alignment, (*) indicates conserved residues, (:) indicates highly similar amino acids, and (.) indicates weakly similar amino acids. Uniprot accession numbers for the proteins are Q63K35 (BipC), E1WAC8 (SipC) and P18012 (IpaC). Domains identified as being involved in actin nucleation (red) or F-actin binding/bundling (blue) are indicated within the alignment and are represented as a diagram below.

**Figure S3**


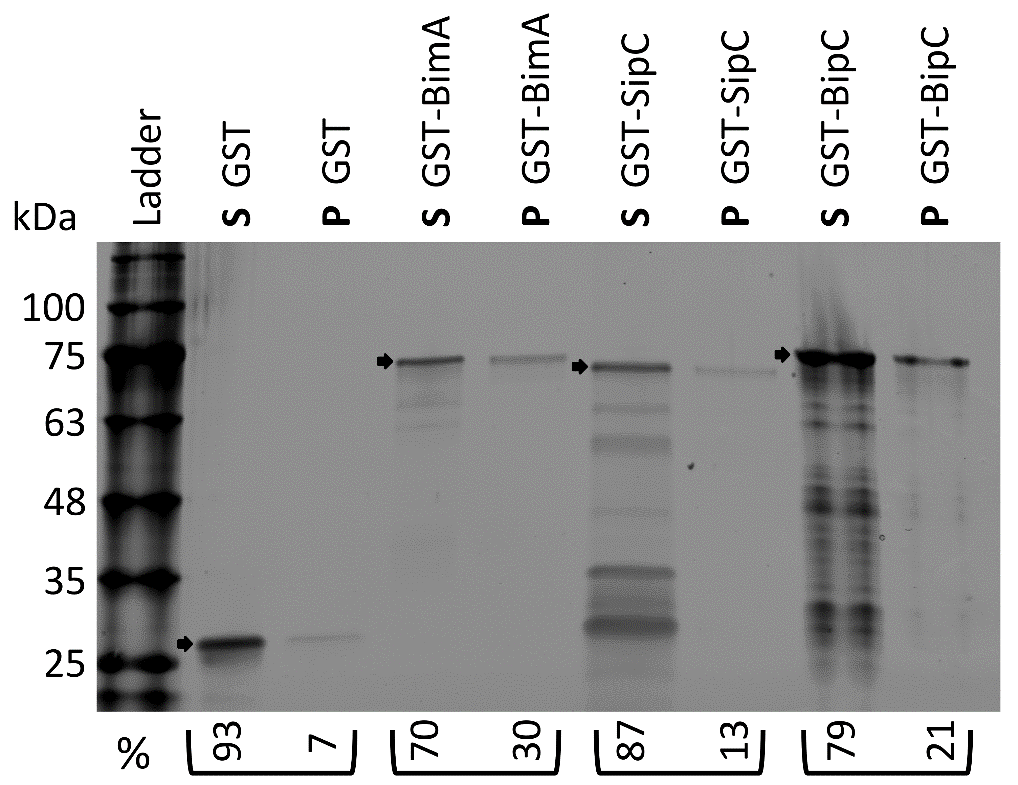


**Figure S3: A representative silver stained gel of the GST proteins sedimented by ultra-centrifugation in the absence of actin.**

GST, GST-BimA_48-384_, GST-SipC or GST-BipC were incubated for 30 minutes without actin. The mixtures were submitted to ultra-centrifugation at 100,000 RCF for 1 hour. The supernatant (**S**) and pellet (**P**) were carefully separated, and denatured in Laemmli buffer. The samples were separated by SDS-PAGE and visualised by silver staining. GST-fusion proteins are indicated by the arrows. The percentage of each GST-fusion protein found either in the supernatant or pellet (as determined by densitometry) is indicated below the corresponding lane (**%**).

**Figure S4**


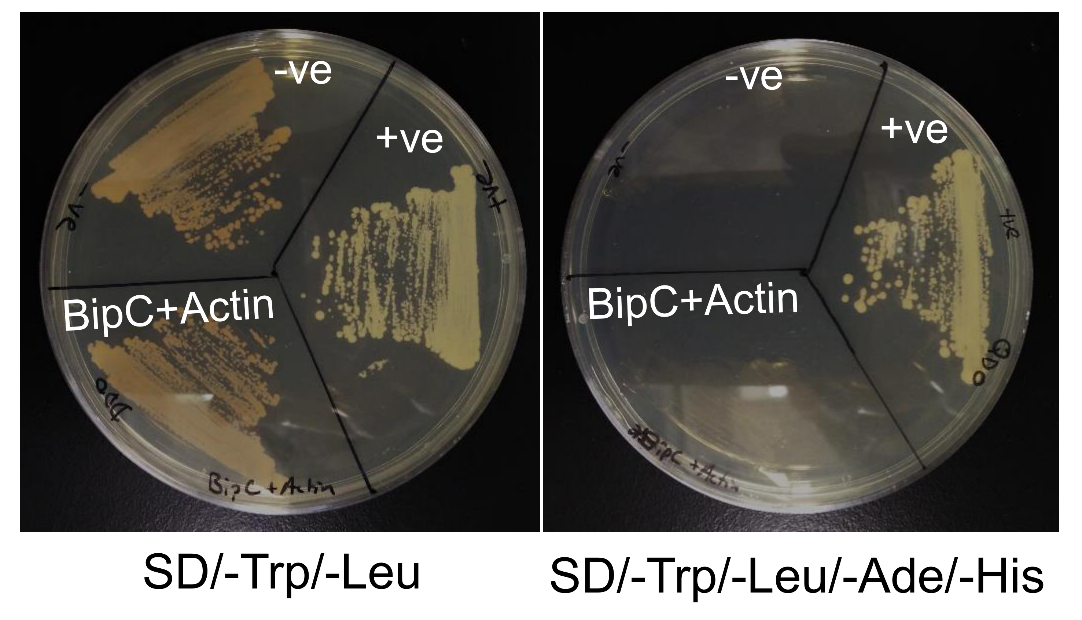


**Figure S4: Yeast two-hybrid assay does not detect an interaction between BipC and actin.**

pGBKT7-BipC and pGADT7-Actin were transformed into *S. cerevisiae* AH109. Strains were plated on SD/-Trp/-Leu and SD/-Trp/-Leu/-Ade/-His plates and incubated at 37^o^C for 5 days. *S. cerevisiae* containing pGBKT7-53 and pGADT7-T was used as a positive control (+ve) and *S. cerevisiae* containing pGBKT7-Lam and pGADT7-T was used as a negative control (-ve) for growth on SD/-Trp/-Leu/-Ade/-His plates.

**References**

Chang J, Chen J, Zhou D. Delineation and characterization of the actin nucleation and effector translocation activities of *Salmonella* SipC. Molecular microbiology. 2005; 55(5):1379-89.

Hayward RD, Koronakis V. Direct nucleation and bundling of actin by the SipC protein of invasive *Salmonella*. The EMBO journal. 1999; 18(18):4926-34.

Maegraith BG and Leithead CS. Melioidosis: a case-report. Lancet. 1964; 283:862-863

Myeni SK, Zhou D. The C terminus of SipC binds and bundles F-actin to promote *Salmonella* invasion. Journal of Biological Chemistry. 2010; 285(18):13357-63.

Paulin SM, Jagannathan A, Campbell J, Wallis TS, Stevens MP. Net replication of *Salmonella enterica* serovars Typhimurium and Choleraesuis in porcine intestinal mucosa and nodes is associated with their differential virulence. Infection and immunity. 2007; 75(8):3950-60.

Sievers F, Wilm A, Dineen D, Gibson TJ, Karplus K, Li W, Lopez R, McWilliam H, Remmert M, Söding J, Thompson JD. Fast, scalable generation of high‐quality protein multiple sequence alignments using Clustal Omega. Molecular systems biology. 2011; 7(1):539.

Stevens MP, Stevens JM, Jeng RL, Taylor LA, Wood MW, Hawes P, Monaghan P, Welch MD, Galyov EE. Identification of a bacterial factor required for actin‐based motility of *Burkholderia pseudomallei*. Molecular microbiology. 2005; 56(1):40-53.

Terry CM, Picking WL, Birket SE, Flentie K, Hoffman BM, Barker JR, Picking WD. The C-terminus of IpaC is required for effector activities related to *Shigella* invasion of host cells. Microbial pathogenesis. 2008; 45(4):282-9.
